# The Role of the Prefrontal Cortex and Functional Connectivity during Maritime Operations: An fNIRS study

**DOI:** 10.1002/brb3.1910

**Published:** 2020-11-04

**Authors:** Shiqi Fan, Eduardo Blanco‐Davis, Jinfen Zhang, Alan Bury, Jonathan Warren, Zaili Yang, Xinping Yan, Jin Wang, Stephen Fairclough

**Affiliations:** ^1^ Intelligent Transport Systems Research Centre Wuhan University of Technology Wuhan China; ^2^ National Engineering Research Centre for Water Transport Safety (WTSC) MOST Wuhan China; ^3^ Liverpool Logistics Offshore and Marine (LOOM) Research Institute Liverpool John Moores University Liverpool UK; ^4^ School of Psychology Liverpool John Moores University Liverpool UK

**Keywords:** Functional Neuroimaging, Neurovascular Coupling, Occupational Safety, Prefrontal Cortex, Spectroscopy, Near‐infrared

## Abstract

**Introduction:**

Watchkeeping is a significant activity during maritime operations, and failures of sustained attention and decision‐making can increase the likelihood of a collision.

**Methods:**

A study was conducted in a ship bridge simulator where 40 participants (20 experienced/20 inexperienced) performed: (1) a 20‐min period of sustained attention to locate a target vessel and (2) a 10‐min period of decision‐making/action selection to perform an evasive maneuver. Half of the participants also performed an additional task of verbally reporting the position of their vessel. Activation of the prefrontal cortex (PFC) was captured via a 15‐channel functional near‐infrared spectroscopy (fNIRS) montage, and measures of functional connectivity were calculated frontal using graph‐theoretic measures.

**Results:**

Neurovascular activation of right lateral area of the PFC decreased during sustained attention and increased during decision‐making. The graph‐theoretic analysis revealed that density declined during decision‐making in comparison with the previous period of sustained attention, while local clustering declined during sustained attention and increased when participants prepared their evasive maneuver. A regression analysis revealed an association between network measures and behavioral outcomes, with respect to spotting the target vessel and making an evasive maneuver.

**Conclusions:**

The right lateral area of the PFC is sensitive to watchkeeping and decision‐making during operational performance. Graph‐theoretic measures allow us to quantify patterns of functional connectivity and were predictive of safety‐critical performance.

## Graphical abstract

fNIRS was used in a ship bridge simulator to study safety‐related performance during watchkeeping and decision‐making. Functional connectivity of the prefrontal cortex was measured. It was found that connection density was reduced during decision‐making while local clustering increased; connectivity measures were also significantly predictive of safety‐related behaviour.

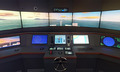

## 1 INTRODUCTION

Human factors are implicated in 75%‐96% of accidents that occur at sea (Fan et al., 2020; Trucco et al., 2008). According to an annual report on marine casualties and incidents issued by the European Maritime Safety Agency, 71% of the factors that contributed to 1170 accident events originated from shipboard operations (EMSA, 2017). It is notable that nontechnical skills (NTS) (Saeed et al., 2016), such as situational awareness (SA) (Stanton et al., 2001) and decision‐making, play a significant role in common types of a maritime accident, such as collisions. For example, a failure to spot another vessel and a failure to correctly estimate speed (of another vessel) are common causes of collisions at sea (Macrae, 2009). The analysis of collision accidents performed by Uğurlu et al. (2015) identified two primary causal pathways, those originating from failures of navigation or maneuvering (e.g., faulty route and wrong maneuver) and those stemming from perception failures (e.g., failures of communication and failure to interpret information correctly). Other analyses of collisions at sea have identified a number of significant precedents, including reduced visibility, misinterpretation of instruments, loss of situational awareness, attention deficits of the officer, and poor intership communication (Chauvin et al., 2013).

Watchkeeping is one of the most significant tasks performed by a desk officer on the bridge (O’Connor & Long, 2011) and is crucial for prevention of collisions at sea. During watchkeeping, officers must observe and record the position of the vessel at regular intervals while paying attention to onboard equipment. In order to sustain high levels of situational awareness, information from radar, visual lookout, and Automatic Identification System (AIS) apparatus are amalgamated into an assessment of the scenario by an officer on the bridge. There are two aspects to watchkeeping activity; one involves sustaining attention to potential obstacles in the vicinity of the vessel. The second incorporates decision‐making and action selection if another vessel is located, and the officer’s vessel is obliged to alter its course. It has been proposed that insufficient watchkeeping accounts for two‐thirds of all collisions at sea (MAIB, 2004). An analysis of collision accidents between vessels and offshore facilities (Sandhåland et al., 2015) identified three distinct categories of error; they were failures to correctly perceive the situation, accurately comprehend the situation, and project the situation into the future. Failures of perception, communication, and decision‐making all play significant roles in a sequence of events known to increase the probability of a collision.

The neuroscience of watchkeeping can be understood from a neuroergonomic perspective (Ayaz & Dehais, 2019; Parasuraman, 2003; Parasuraman & Rizzo, 2008) with respect to the neuroscience of vigilance and action selection. The task of sustaining attention over a long period of time has been extensively studied in human factors psychology (Davies & Parasuraman, 1982; Hancock, 2017; Mackworth, 1948; Warm, 1984). It is known that sustaining attention can be particularly challenging when the task is monotonous and intellectually undemanding (Parasuraman, 1984; Robertson & O’Connell, 2010), as is the case during watchkeeping at sea. It has been argued that availability of those attentional resources that are necessary to sustain attention on a specific task declines over time and reduces the quality of attention focused on the task (Warm et al., 2008). Alternatively, it has been claimed that the monotonous and uninteresting nature of vigilance tasks leads inevitably to disengagement from the task at hand (Smallwood & Schooler, 2006); see review by Fortenbaugh et al. (2017) for recent discussion of both perspectives. With respect to those areas of the brain that are implicated during sustained attention, early work on neuroimaging suggested that vigilance performance was associated with increased activation in the right prefrontal cortex (Cohen et al., 1988, 1992; Coull et al., 1998; Lewin et al., 1996; Parasuraman et al., 1998). The meta‐analyses performed by Langner and Eickhoff (2013) identified neurological clusters in the right hemisphere associated with sustained attention and the duration of a vigil, which included anterior sulcus, inferior frontal sulcus (BA46), middle/anterior thalamus, precentral sulcus, inferior parietal lobule, posterior inferior frontal gyrus, cerebellum, and temporoparietal junction. The same analysis also identified an association between sustained attention and activation of the right midlateral area of the prefrontal cortex (BA9, BA46), particularly tasks with a variable (as opposed to a fixed) schedule of event occurrence, where no overt response was required; more importantly, in addition, this region was implicated across multiple modalities of stimuli, for example, visual and auditory.

The second aspect of watchkeeping concerns those cognitive control processes of decision‐making and action preparation/selection, which are activated when another vessel has been located and the potential for a collision is apparent. Koechlin et al. (2003) described a hierarchical model of cognitive control, wherein the selection of motor actions in response to task stimuli (sensory control) is informed by existing stimulus–response associations for the situational context (contextual control), which, in turn, are determined by recall of previous experience (episodic control). This model hypothesized that sensory control was localized to the motor cortex. In contrast, contextual and episodic levels of control were associated, respectively, with bilateral activation of caudal (BA44/45) and rostral (BA46) regions of the lateral prefrontal cortex (LPC). This model was further developed by Koechlin and colleagues (Domenech & Koechlin, 2015; Koechlin & Summerfield, 2007), who proposed two methods of arbitration for executive control: (a) a peripheral system located in the premotor/caudal/orbitofrontal regions for action selection based on perceptual cues and reward values that are stable and (b) a core system incorporating regions of the ventromedial, dorsomedial, lateral, and polar PFC that adjust between exploitation/adjustment of previously learned behavioral sets and exploration/creation of a new behavioral set. According to this model, the possibility of a obtaining a desirable outcome via a specific behavioral task set is explored via the ventromedial region of the PFC. If there is a mismatch, the system reverts to the dorsomedial and lateral regions of the PFC to either create a new task set or select an alternative task set with a greater chance of a desirable output; for elaboration of model and further explanation, see Koechlin (2016).

Both sustained attentional and cognitive control are fundamental to the activity of watchkeeping during ship operations and associated with distinct patterns of activation within the PFC. Functional near‐infrared spectroscopy (fNIRS) (Ferrari & Quaresima, 2012; Scholkmann et al., 2014) has been used widely to study neurophysiological activation during operational performance (Ayaz et al., 2012, 2013), both during task simulation (Gateau et al., 2015; Modi et al., 2018; Unni et al., 2017) and real‐world environments (Dehais et al., 2018; Foy et al., 2016). It is often reported that increased cognitive demand is associated with an increased level of oxygenated hemoglobin (HbO) in the prefrontal cortex (Causse et al., 2017; Fairclough et al., 2018). Alternatively, one can utilize fNIRS to index changes in functional connectivity under different task conditions by measuring the degree of correlation between HbO values collected simultaneously at various locations across the cortex. For example, Verdière et al. (2018) utilized a number of connectivity features (e.g., covariance, correlation, and wavelet coherence) to successfully distinguish low from high levels of mental workload during a simulated aircraft landing scenario. Frontal connectivity across the PFC has also been found to increase with cognitive demand using laboratory tasks (Baker et al., 2018; Racz et al., 2017; Sun et al., 2019). In addition, global indices of connectivity (e.g., wavelet phase coherence) between the left PFC and sensorimotor areas were found to decline during a vigilance task (Wang et al., 2016). Similarly, a study of simulated driving reported reduced connectivity between PFC and motor cortex over an hour of sustained performance (Xu et al., 2017). A decline of connectivity in association with task‐related fatigue was also reported in maritime operators with respect to bilateral activity in the PFC (Bu et al., 2016). Some researchers have deployed metrics derived from graph theory (Welton et al., 2015) in order to describe connectivity networks revealed by fNIRS data (Einalou et al., 2017; Racz et al., 2017). This approach is utilized in order to describe functional brain networks as connectomes, capable of describing the development of mental fatigue (Qi et al., 2019) or the level of expertise of a specific operator (Deligianni et al., 2020).

A study was conducted in a ship bridge simulator to explore neurophysiological activation in the prefrontal cortex when participants performed watchkeeping activity that was divided into two phases: sustained attention and decision‐making/action selection. It was hypothesized that the right lateral region of the PFC (e.g., BA9 and BA46 on right side) would be activated during sustained attention. Action selection/preparation during the decision‐making phase of the simulation will bilaterally activate the caudal and rostral areas of the PFC as participants appraise the situational context of the scenario. It was also predicted that neurovascular activation and functional connectivity across the PFC would decline over the period of sustained attention. It was anticipated that experienced seafarers with greater number of hours at sea and higher level of qualification would demonstrate greater situational awareness and efficient decision‐making and maintain a greater safety margin, that is, the target vessel will be spotted earlier, and an evasive maneuver would be made at greater distance from the target vessel. In addition, it was hypothesized that experienced individuals would exhibit greater neural efficiency, that is, reduced activation of the PFC when performing the simulation compared to inexperienced participants (Causse et al., 2017). Half of the participants were also required to perform an additional distraction task in conjunction with watchkeeping. This requirement to regularly report the position of the vessel was designed to increase mental workload and activation of the PFC, and degrade performance outcomes (i.e., spot and respond to target vessel at a lower distance).

## 2 METHOD

### 2.1 Experimental design

The independent variables for the study were as follows: experience of participant, mental workload (watchkeeping vs. watchkeeping + ship position reporting), and the periods of the task simulation (sustained attention vs. decision‐making). Participant experience and mental workload served as between‐participant variables, whereas the periods of the simulation constituted a within‐participant manipulation.

### 2.2 Participants

Forty participants were recruited from the Nautical Institute London Branch and Liverpool John Moores University’s Maritime Centre. Participants were divided into two groups of 20 individuals based on their Standards of Training, Certification, and Watchkeeping (STCW) qualification and seafaring experience. The group of experienced seafarers were all qualified as MM (Master), CM (Chief Mate), and OOW (Officer of the Watch), this group included one female, their average age was 44.6 yrs (sd = 15.5), and they had an average of 213.4 months (sd = 188.8) experience at sea. The inexperienced group were qualified as AB (Able Seaman) or cadets; this group included two females, had an average age of 25 yrs (sd = 5.4), and had acquired 27.2 months (sd = 30.5) experience at sea. Exclusion criteria included history of head injury, high blood pressure, anxiety, or currently taking medication for anxiety. The experimental protocol for the study was approved by the institutional ethics committee prior to data collection.

### 2.3 Ship bridge simulator and scenario

**Figure 1:**
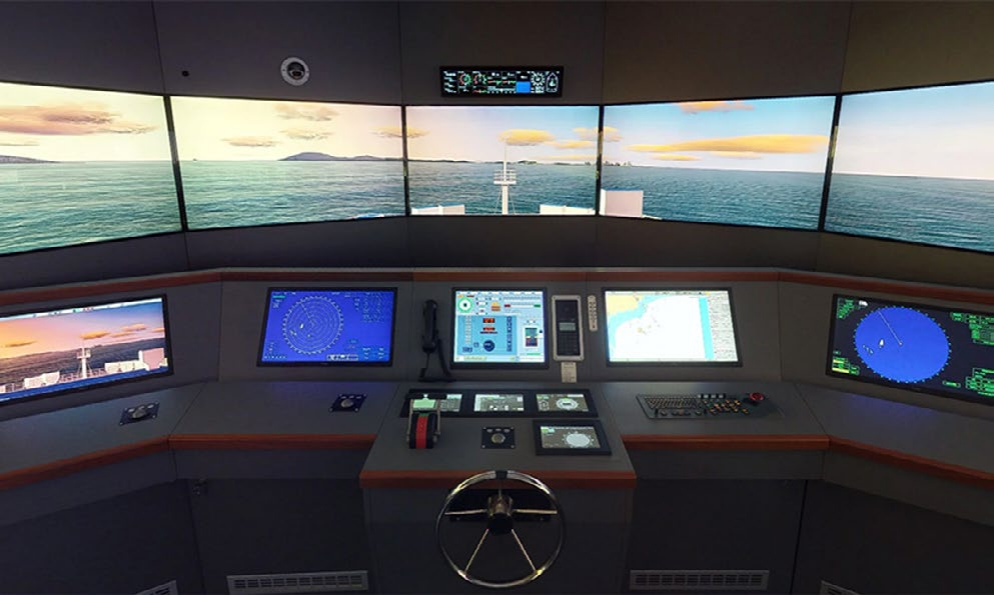
View of the participant in the ship bridge simulator

The experiment took place in a ship bridge simulator (Transas) fitted with instrument panels located at Liverpool John Moores University. An illustration of the participant view of the facility is provided in Figure 1. The Transas simulator is configurable for specific ship types using ship modeling software which manages the simulation environment, allowing for positioned interactive tides, currents, geographically variable wind, and sea, and changing conditions such as light, visibility, fog, and rain. The bridge simulator can deliver a 360° field of view, but the display was constrained to a 180° field of view for the purpose of the current study for two reasons: (a) The scenario involved watchkeeping in the forward view only, and (b) we wished to avoid significant lateral movement of the head and upper body to minimize artifacts in the fNIRS data.

**Figure 2:**
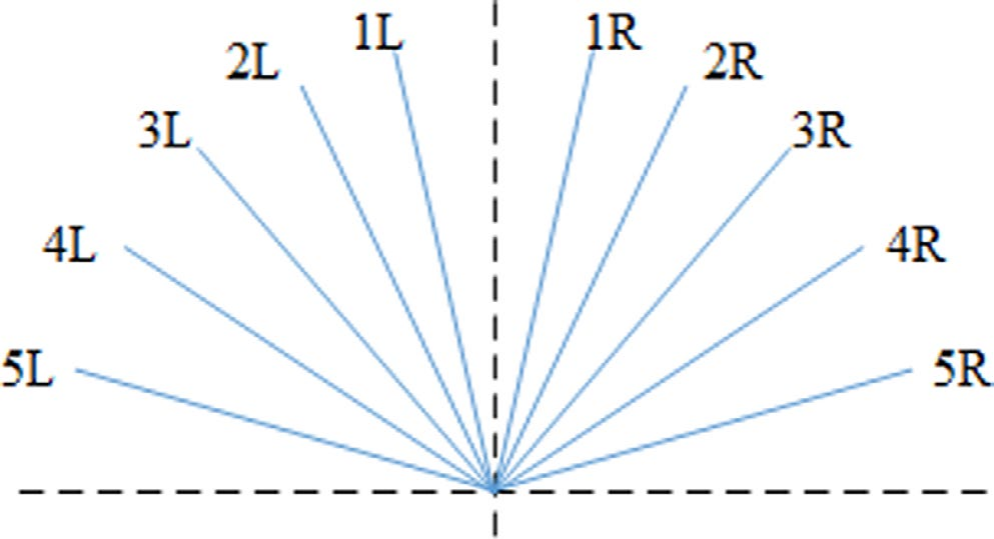
The ten potential positions of the target vessel in the 180° field of view relative to sightline of participant (vertical dotted line)

The task scenario was designed to occur along a north/south axis to better accommodate a realistic reporting system. All participants were required to keep watch over 180° field of view of the open sea. This watchkeeping period was terminated when participants spotted a “target” vessel that appeared randomly at one of 10 locations in the field of view (see Figure 2). The target vessel was the only other ship on the ocean in the whole of the task simulation.

Participants were required to push the button when they spotted the target vessel, the approximate location of which was recorded by the staff in the control room. On average, the duration of this watchkeeping/sustained attention phase of the task was 19 min:42 sec. The distance in nautical miles between the target vessel and the participants’ ship when the button was pressed was captured as a dependent variable. The target vessel approached the participants’ ship on a course that would lead to a collision if a change of course was not made; the speed of approach from the target vessel was approximately 15–20 knots. Once participants had spotted the target vessel, the scenario enters a decision‐making/action selection phase where participants had to visually monitor the course and speed of the vessel in order to assess the risk of collision. This decision‐making/action selection phase was terminated when participants turned the helm on the bridge (Figure 1) to change course and make an evasive maneuver; the experiment also ended at this point. On average, all participants made an evasive maneuver at 24 min: 26 sec; the distance in nautical miles between target vessel and participants’ ship when the maneuver was made was also recorded as a dependent variable.

### 2.4 distraction task

In addition to the scenario described in the previous section, half of the participants (10 experienced and 10 inexperienced) were required to perform a reporting task, which served as a distraction and was based on existing procedures. Participants were required to monitor the ECDIS (Electronic Chart Display and Information System) in order to make a verbal report of the position of their vessel, that is, numeric coordinates of ships’ current position. Participants in the distraction group made this verbal report whenever their vessel crossed a predetermined reporting point, for example, they must report the ship position when the vessel crossed each minute of latitude as displayed on the ECDIS. With their vessel steering a northerly course at a constant speed of 20 knots, this task amounted to a requirement to make a report every three minutes.

### 2.5 Subjective mental workload

The NASA Task Load Index (TLX) (Hart & Staveland, 1988) was administered at the end of the experiment. This is a self‐assessed questionnaire constructed upon six 10‐point scales: Mental Demand, Physical Demand, Temporal Demand, Performance, Effort, and Frustration.

### 2.6 fNIRS device and montage

**Figure 3:**
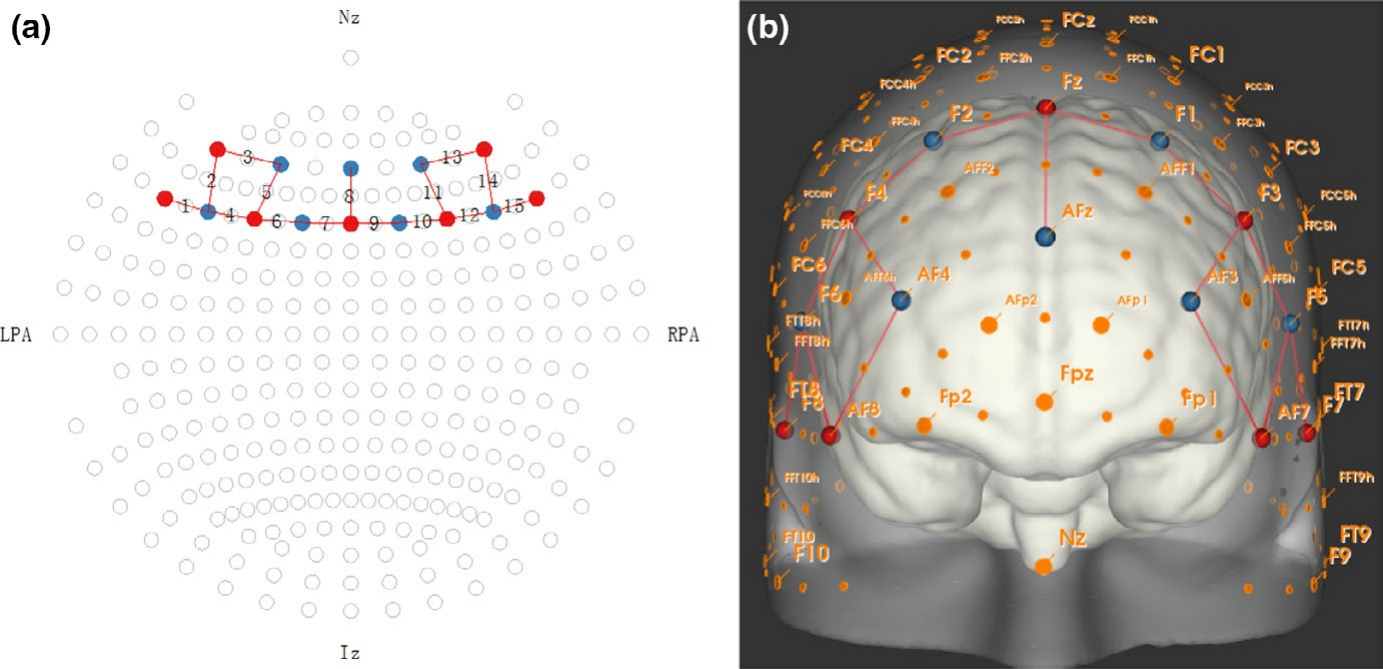
Illustration of fNIRS montage: (a) 2D montage (b) 3D montage. Red = emitter, blue = detector

The NIRSport 88 (NIRx Medical Technologies LLC, USA) fNIRS device was utilized to capture neurovascular measures of activation. This device records optical density data at a frequency of 8.9Hz and consists of 8 sources and 8 detectors. The device was configured to detect deoxygenated and oxygenated hemoglobin at wavelengths of 760 nm and 850 nm. The NIRSite software was used to construct a montage of 15 channels over the prefrontal cortex (Figure 3).

### 2.7 fNIRS analysis I: average HbO

The fNIRS data (15 channels × 2 wavelengths) were preprocessed using nirsLAB software. Raw data were checked for discontinuities and spikes and interpolation applied where necessary via the nirsLAB software. A low‐pass filter was subsequently applied in order to reduce high‐frequency instrument noise and physiological noise such as fast cardiac oscillations (e.g., heartbeat 1 ~ 1.5Hz) with the frequency of 0.4Hz. Changes in oxygenated hemoglobin (HbO) and deoxygenated hemoglobin (Hbb) were calculated using the modified Beer–Lambert law (mBLL) using differential path factor (DPF) of 7.25 (760nm) and 6.38 (850nm). The HbO and Hbb data were subjected to a correlation‐based transformation, the correlation‐based signal improvement (CBSI) (Cui et al., 2010) that forces HbO and Hbb to be negatively correlated. As Hbb is transformed into the inverse of HbO after this point, that is, correlations between HbO and Hbb varied between −0.78 and −0.98 after application of the CBSI, only HbO data were used in the subsequent analyses, and these data are relabeled as CBSI_HbO to differentiate them from original values calculated using the mBLL.

In order to create ANOVA models for statistical testing, 15 channels of CBSI_HbO were divided into three regions of interest: left lateral PFC (channels 1‐5), central PFC (channels 6‐10), and right lateral PFC (channels 11–15) (see Figure 3 and Table 1). The placement of the optodes and corresponding areas from the 10/20 system and Brodmann’s areas are described in Table 1. The average CBSI_HbO for each region was calculated and used in the ANOVA model. In addition, for the purposes of statistical testing, the sustained attention portion of the task simulation (before the ship was located) was divided into four periods of equal duration for each participant (watch1, watch2, watch3, and watch4). Once the ship had been spotted, the subsequent decision‐making phase of the simulation was divided into two periods of equal duration (decision2 and decision2). Values of average CBSI_HbO were calculated for each region of interest for watch1‐4 and decision1‐2 for testing in ANOVA models.

**Table 1:** Placement of optodes by ROI, channel number, 10/20 system, and Brodmann’s areas (see also Figure 3)

ROI	Optode/channel	10/20 System	Brodmann
1 (Left)	1	F5/F7	47/46
	2	AF7/F5	46
	3	AF7/AF3	46/9
	4	F3/F5	46/8
	5	F3/AF3	8/9
2 (Central)	6	F1/F3	8
	7	Fz/F1	8
	8	Fz/AFz	8/9
	9	Fz/F2	8
	10	F2/F4	8
3 (Right)	11	F4/AF4	8/9
	12	F4/F6	8/46
	13	AF4/AF8	9/46
	14	AF8/F6	46
	15	F6/F8	46/45

### 2.8 fNIRS analysis II: functional connectivity

The analysis of functional connectivity used in the current study was based on the procedure described by Racz et al. (2017). Unlike those authors, our analysis of functional connectivity was based upon a matrix of partial correlation coefficients calculated between each available channel of CBSI_HbO, that is, partial correlation coefficients represent association between two channels of CBSI_HbO while controlling for the effect of the other 13 channels (Akın, 2017; Dadgostar et al., 2016). A matrix of partial correlation coefficients (partial r) was calculated for all 15 channels of CBSI_HbO for each of the six periods of the simulation (watch1, watch2, watch3, watch4, decision2, and decision2) for each participant.

A process of thresholding was applied to each matrix of partial r values in order to construct a binary functional connection network. The first step of this analysis was to remove any partial r values that fell below zero to consider only positive associations. A criterion level of 0.28 was selected in order to remove weak or spurious levels of correlation; this value represents the critical value for a one‐tailed test of Pearson’s coefficient at *p* < .05 for N = 40. This thresholding process converted the original matrices of partial correlations into binary adjacency matrices suitable for graph‐theoretic analyses.

Measures of connection density (D) and local clustering coefficient (C) were calculated for each participant per period of the experiment on the basis of the binary matrices (Racz et al., 2017). The connection density of a network is the fraction of the existing connections to all possible connections, which is calculated as follows:

$$D=\frac{1}{2n\left(n‐1\right)}\sum_{i\in n}\sum_{j\in n}a_{\mathrm{ij}}$$

where *n* is the number of channels in the network, and *a_ij_* equals 1 if there is a connection between channels *i* and *j*, 0 otherwise. In summary, Density describes the overall level of connectivity between all nodes in the network. A clustering coefficient was also calculated for the network. This index of local clustering quantifies the proportion of neighbors to a node which are also neighbors of one other (Watts & Strogatz, 1998), that is, reflecting the number of triangles around the given node (Rubinov & Sporns, 2010). The clustering coefficient was calculated as follows:

$$C=\frac{1}{n}\sum_{i\in n}\frac{1}{k_{i}\left(k_{i}‐1\right)}\sum_{j,h\in n}a_{\mathrm{ij}}a_{\mathrm{ih}}a_{\mathrm{jh}}$$

where *k_i_* is the degree of channel *i*, and C is how the neighboring channels in the network form connected groups. These functions were obtained from the Brain Connectivity Toolbox (Rubinov & Sporns, 2010). Measures of D and C were calculated per participant for each period of the task and subjected to statistical testing via ANOVA.

### 2.9 Experiment procedure

Participants arrived at the simulator and were required to read the Participant Information Sheet and provide signed consent. The participant subsequently took a seated position at the helm (Figure 1) of the vessel and completed a short familiarization session to check that they understood how to operate the helm and read data from the displays (Figure 1). All participants were told that they would be asked to monitor their vessel, which was on a fixed course in a shipping lane and completes a watchkeeping task; they were provided with no other information. Participants in the distraction group received additional instructions pertaining to the distraction task (as described in section 2.4). Each participant was fitted with the NIRx Sport head cap, which was placed using nasion and inion as anatomical points for longitudinal placement and the pre‐auricular points above the ears for lateral placement. Once the head cap had been fitted, the quality of the signal was assessed. The study did not commence until acceptable signal quality was obtained from all 15 channels. Once the signal quality had been approved, the experimenter took a seated position behind the participant and the participants conducted the task scenario as described in section 2.3, commencing with the watchkeeping task, that is, they would navigate in the open water under watchkeeping task and make an evasive maneuver if necessary. Participants were instructed to press a button on the console if they spotted another vessel; if this occurred, they were instructed to monitor the course and position of the other vessel and change course if there was any possibility of a collision. The task scenario ended when participants made their evasive maneuver to avoid the target vessel. Participants in the distraction group received additional instructions pertaining to that task. When the experiment had been completed, all participants completed the TLX questionnaire. Participants were subsequently debriefed and thanked for their time, and received a gift voucher for their time.

## 3 RESULTS

The results section is divided into four sections: behavioral data, subjective mental workload, average level of CBSI_HbO at specific sites, and functional connectivity. Data are subjected to statistical analyses via ANOVA, ANCOVA, and MANOVA models using SPSS v.26. Outliers were defined as any data point lying more than 3 standard deviations from the mean for that “cell” in either a positive or negative direction. For those models with a repeated‐measures component, sphericity was tested using Mauchly’s test and the Greenhouse–Geisser adjustment was performed.

### 3.1 Behavioral data

Behavioral data were derived from two responses required from all participants; they were required to (1) spot another vessel (target spotted) and to (2) change their course as an evasive maneuver to avoid collision (course change). Distance in nautical miles was calculated when the target vessel was spotted (1) and when a course change was made (2). The analysis of the target spotted distances took the form of a univariate ANCOVA (Experience x distraction) with the relative position of the target vessel functioning as a covariate (Figure 2). This model failed to reveal any significant effects of Experience [*F*(1,40) = 0.58, *p *= .45], distraction [*F*(1,40) = 0.47, *p* = .50], Target Location [F(1,40) = 0.59, *p* = .45], or any interaction (see Table 2 for descriptive statistics). The analyses of distance when course was changed was performed as an ANCOVA (Experience x distraction), which revealed a significant effect of Experience [F(1,40) = 4.53, *p* = .04, η_p_^2^ = 0.11], no significant effect for distraction [F(1,40) = 2.75, *p* = .11], and no significant interaction. Inspection of means (Table 3) revealed that experienced participants performed the course change at greater distance from the target vessel compared to inexperienced participants.

**Table 2:** Means and standard deviations for distance (in nautical miles) from participant ship to target vessel when the latter was spotted (*N* = 40)

inexperienced	distraction	4.45 [1.27]	4.51 [1.16]
	No distraction	4.57 [1.10]	
experienced	distraction	4.60 [1.03]	4.77 [0.94]
	No distraction	4.94 [0.86]	

**Table 3:** Means and standard deviations for distance (in nautical miles) from participant ship to target vessel when course change occurred (*N* = 40)

inexperienced	distraction	1.80 [1.37]	1.85 [1.43]
	No distraction	1.90 [1.56]	
experienced	distraction	2.10 [1.26]	2.76 [1.37]
	No distraction	3.41 [1.19]	

### 3.2 Subjective mental workload

TLX data were analyzed via a 2 (experienced/inexperienced) x 2 (distraction/no distraction) x 6 (TLX factor) MANOVA. This analysis revealed a main effect of distraction with respect to Temporal Demand (“How much time pressure?”) [F(1, 36) = 3.89, *p* = .05, η_p_^2^ = 0.10], that is, participants in the distraction group perceived a higher level of temporal demand; see Table 3 for descriptive statistics. It was also apparent that experienced participants rated the quality of their Performance as greater than that in the inexperienced group [*F*(1, 36) = 9.19, *p* < .01, η_p_^2^ = 0.20]; in addition, subjective levels of Effort were lower for experienced compared to inexperienced participants [*F*(1, 36) = 5.31, *p* = .03, η_p_^2^ = 0.13]. All descriptive statistics are presented in Table 4, and there were no other significant main effects or interactions in the MANOVA model.

**Table 4:** Descriptive Statistics for NASA Task Load Index ratings of subjective mental workload (*N* = 40)

		distraction	No distraction	Mean
MENTAL Demand	inexperienced	6.50 [1.65]	7.50 [2.53]	7.00 [2.09]
	experienced	6.30 [2.16]	6.20 [2.49]	6.25 [2.33]
	Mean	6.40 [1.89]	6.70 [2.49]	
PHYSICAL Demand	inexperienced	2.30 [1.34]	3.10 [2.08]	2.70 [1.71]
	experienced	2.40 [2.01]	2.20 [1.14]	2.30 [1.58]
	Mean	2.35 [1.89]	2.65 [1.69]	
TEMPORAL Demand	inexperienced	5.20 [1.87]	4.50 [1.96]	4.85 [1.92]
	experienced	4.90 [2.47]	3.10 [1.66]	4.00 [2.07]
	Mean	5.05 [2.14]	3.80 [1.91]	
PERFORMANCE	inexperienced	7.80 [1.23]	8.10 [1.37]	7.95 [1.30]
	experienced	9.00 [0.82]	9.10 [1.10]	9.05 [0.96]
	Mean	8.40 [1.19]	8.60 [1.32]	
EFFORT	inexperienced	6.40 [1.27]	6.40 [2.68]	6.40 [1.98]
	experienced	5.70 [1.83]	4.20 [1.93]	4.95 [1.88]
	Mean	6.05 [1.57]	5.30 [2.54]	
FRUSTRATION	inexperienced	2.70 [1.95]	3.10 [1.97]	2.90 [1.98]
	experienced	3.00 [1.56]	2.50 [1.84]	2.75 [1.96]
	Mean	2.85 [1.73]	2.80 [1.88]	

### 3.3 fNIRS Data I: Average CBSI_HbO

Inspection of fNIRS data indicated portions of missing data from four participants due to equipment failure during the study, who were not included in these analyses; the remaining numbers in each experimental group were as follows: experienced/no distraction: 9; experienced/distraction: 9; inexperienced/no distraction: 9; and inexperienced/distraction: 10. fNIRS data were averaged for each channel and divided into three regions of interest (ROI) corresponding to the left lateral, central, and right lateral areas of the PFC. CBSI_HbO data for each ROI were subjected to a 2 (experienced/inexperienced) x 2 (distraction/no‐distraction) x 6 (task period: watch1, watch2, watch3, watch4, decision2, and decision2) ANOVA.

**Figure 4:**
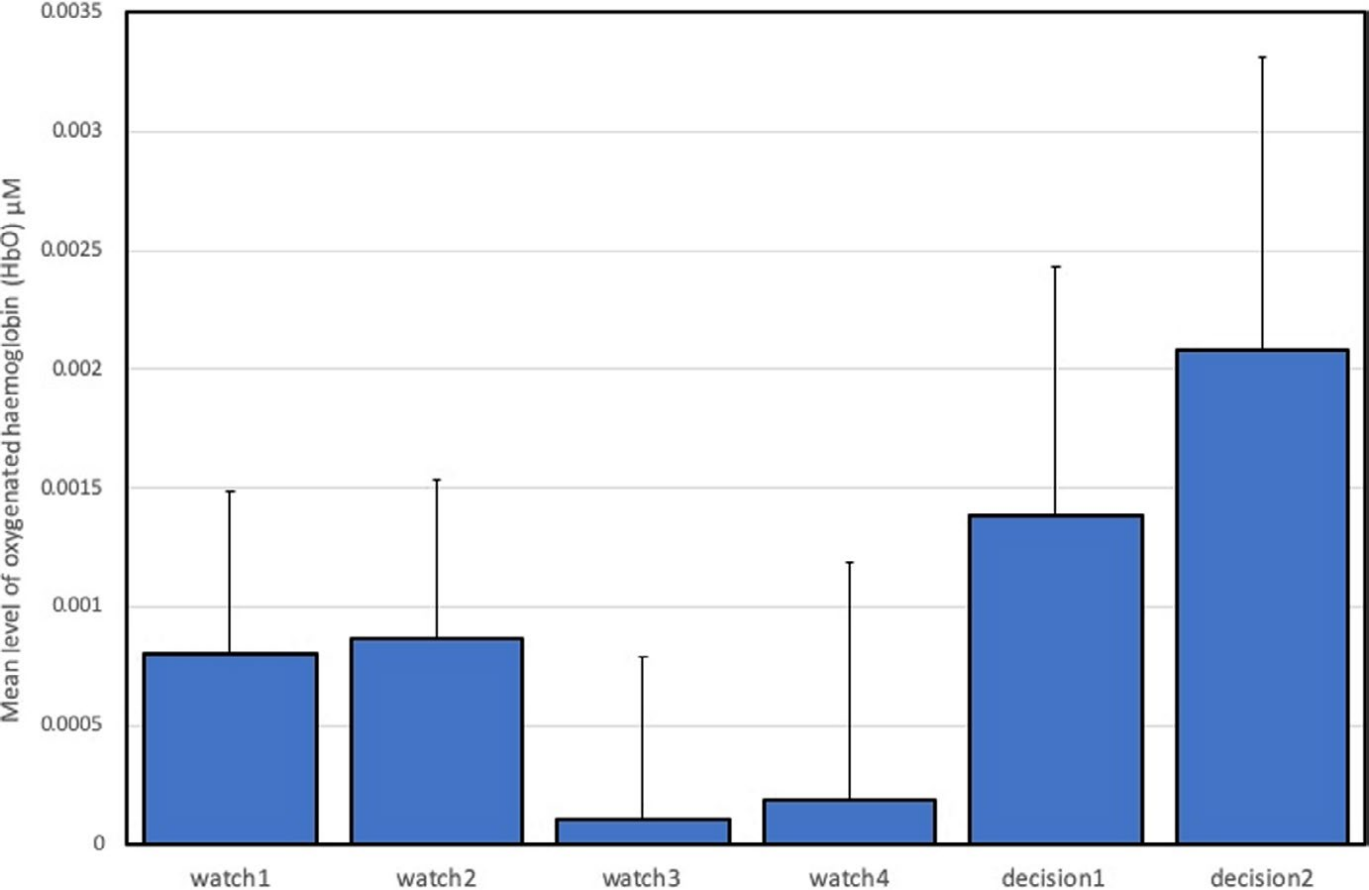
Mean CBSI_HbO and standard error during all task periods for right lateral ROI (*N* = 38)

Analyses of left lateral and central ROI failed to indicate any statistically significant main effects or interactions. However, the analysis of CBSI_HbO data from the right lateral ROI revealed a significant main effect for task period [*F*(5,30) = 3.76, *p* = .02, η_p_^2^ = 0.4], as well as significant interactions between distraction x task period [*F*(5,30) = 3.99, *p* < .01, η_p_^2^ = 0.43] and Experience x task period [*F*(5,30) = 2.30, *p*=.05, η_p_^2^ = 0.27]. Post hoc testing revealed that average CBSI_HbO at the right lateral ROI was significantly lower during watch3 and watch4 than all other periods (*p*<.05); this effect is illustrated in Figure 4.

**Figure 5:**
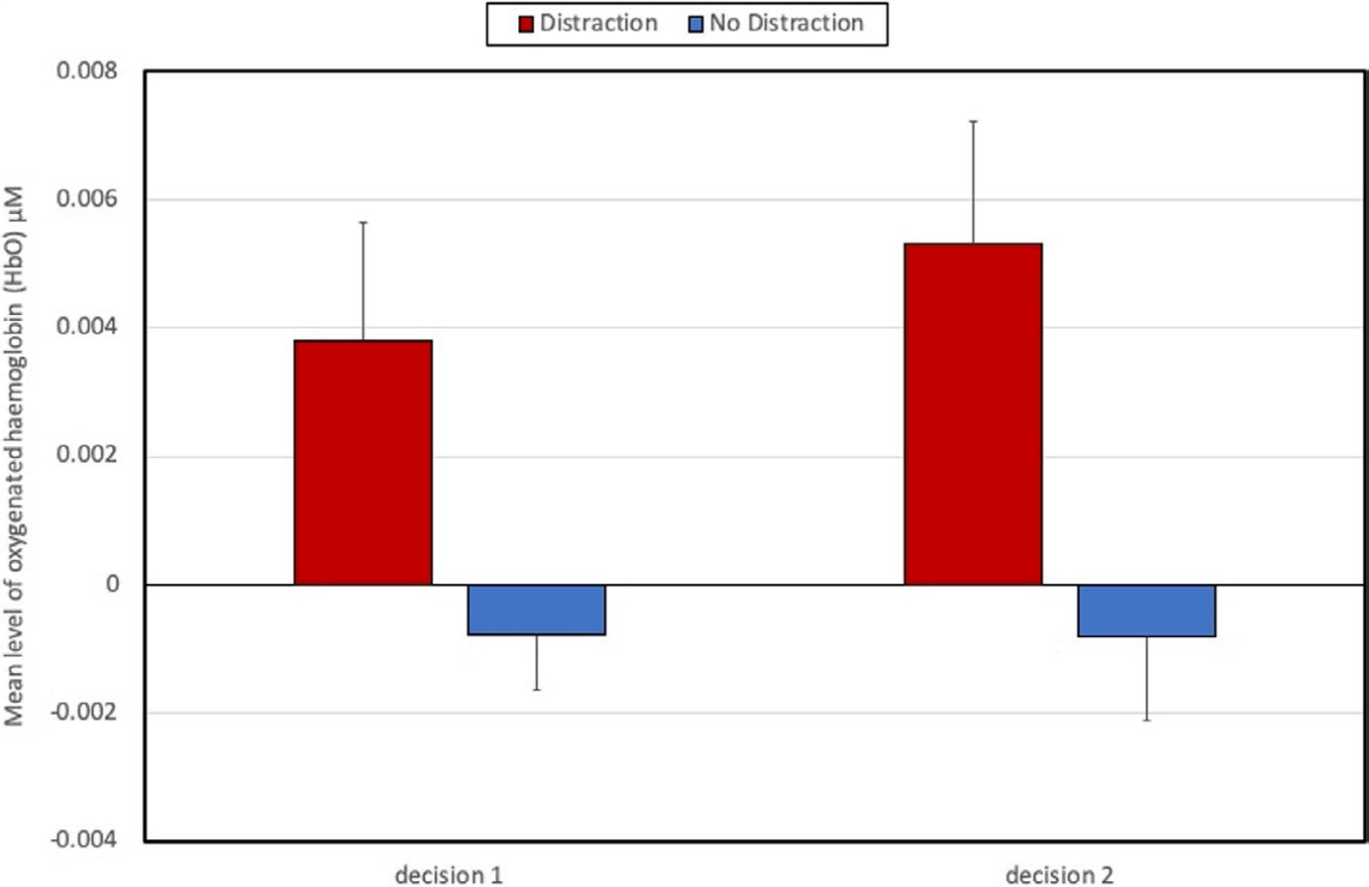
Average CBSI_HbO and standard error in the right lateral ROI for task period x distraction Interaction (*N* = 38)

A number of post hoc t tests were conducted to analyze the two significant interaction effects at the right lateral ROI. It was found that average CBSI_HbO was significantly higher for participants who performed the distraction task during the two periods of decision‐making that occurred once the ship had been spotted: decision1 [t(36)=2.17, *p*=.04] and decision2 [t(36)=2.69, *p*=.02]. This effect is illustrated in Figure 5.

**Figure 6:**
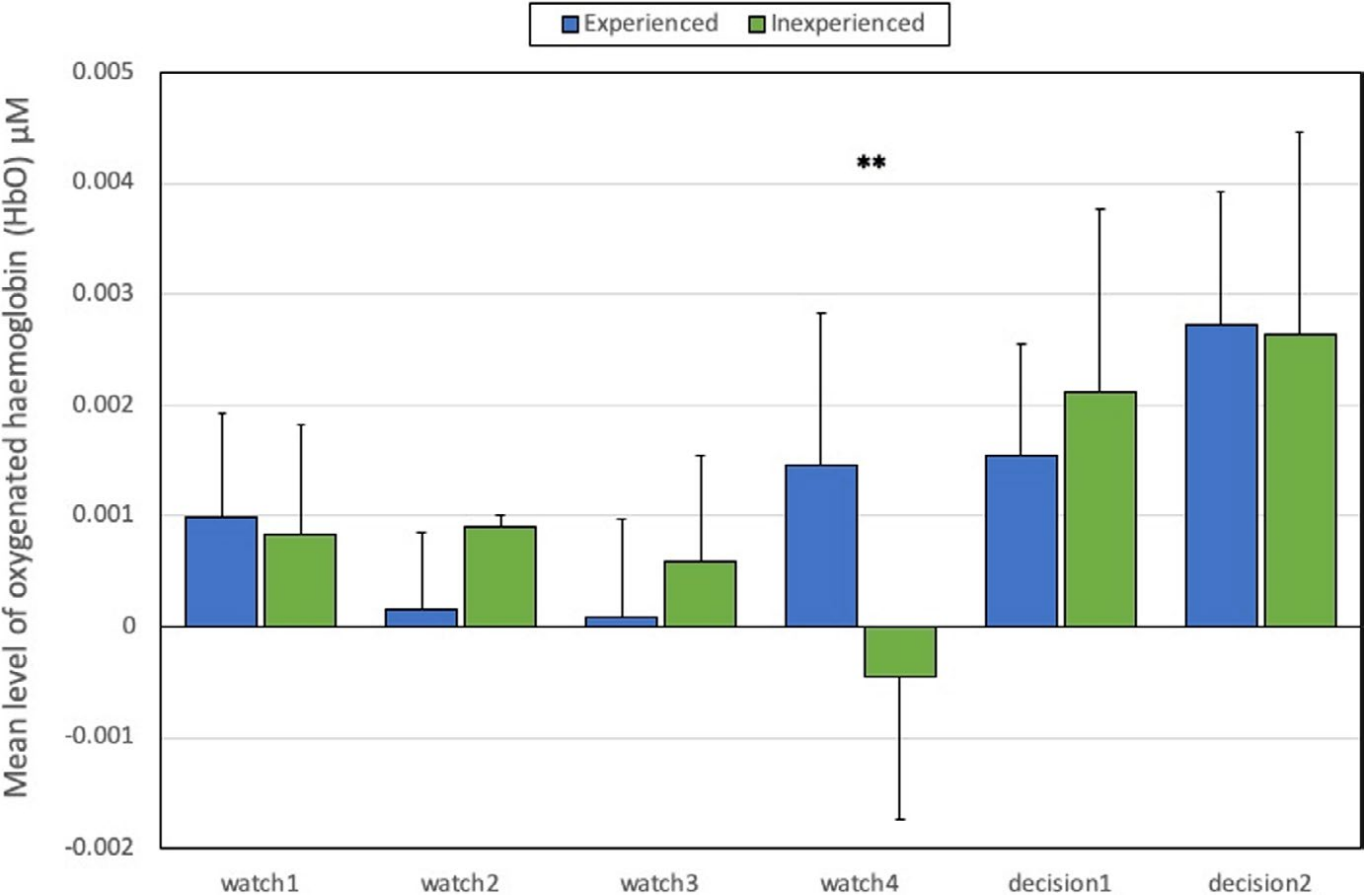
Average CBSI_HbO and standard error in the right lateral ROI for task period x Experience Interaction (*N* = 38). Note: ** = significant difference at *p* < .01

The interaction effect between Experience x task period was also explored using t tests. These tests revealed that average CBSI_HbO was higher for experienced participants at the right lateral ROI, but only during the fourth period of watchkeeping (watch4) when the approaching ship was spotted [*t*(36)=2.78, *p*<.01]. This interaction is illustrated in Figure 6.

### 3.4 Functional connectivity

**Figure 7:**
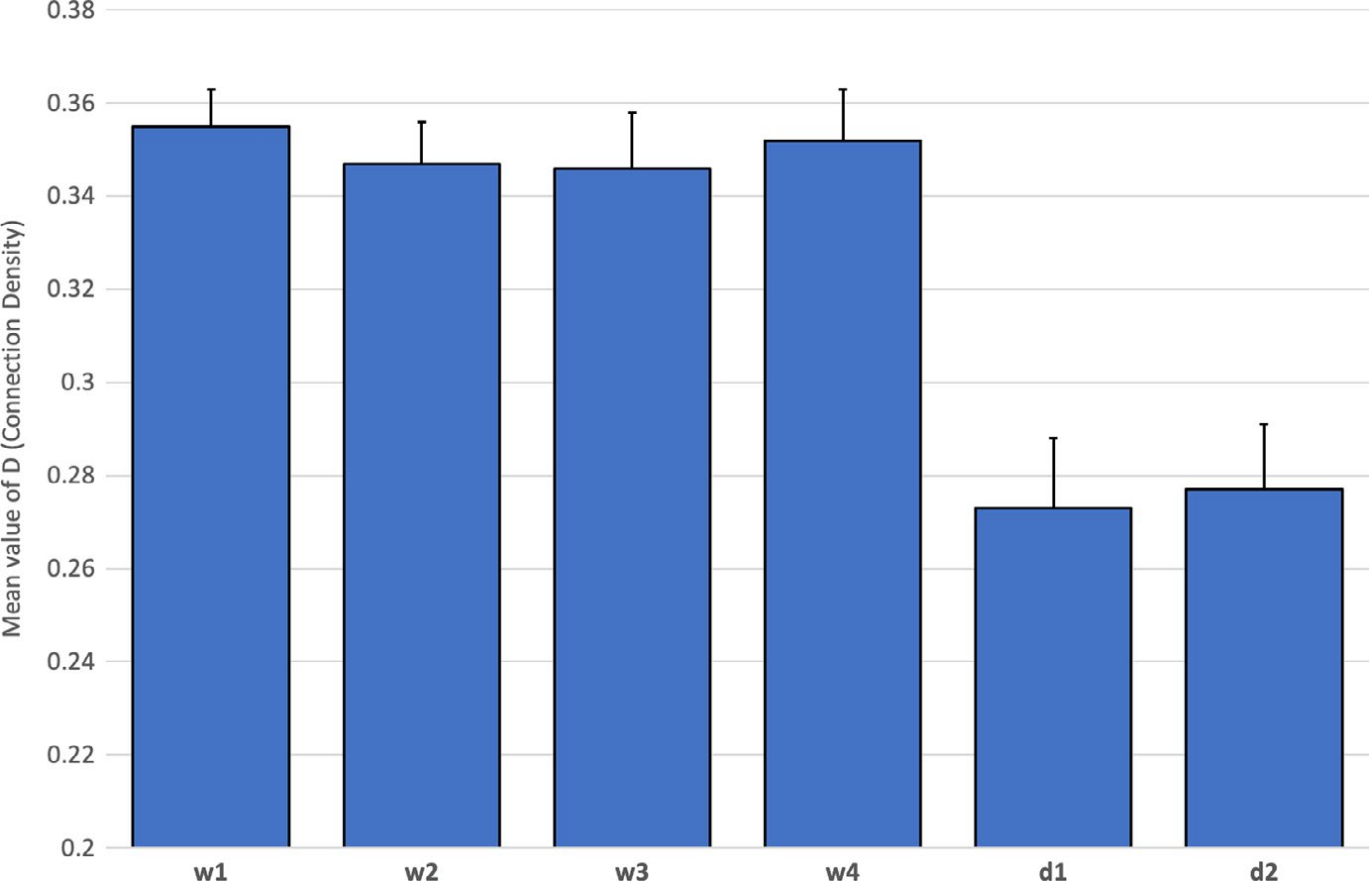
Average levels of D (connection density) with standard error across all fNIRS channels for six periods of the task (*N* = 36)

**Figure 8:**
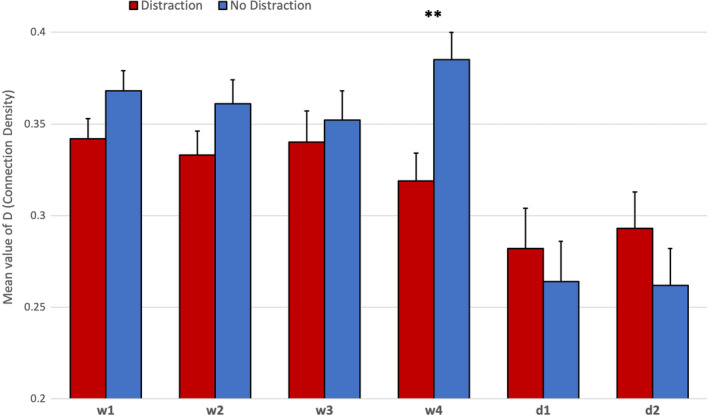
Interaction between distraction group x task periods for mean D (connection density) with standard error across all fNIRS channels for six periods of the task (*N* = 36). Note: ** = significant difference at *p* < .01

A 2 (experienced/inexperienced) x 2 (distraction/no distraction) x 6 (task period) ANOVA was conducted on the measure of connection density (D). This model revealed a significant main effect for task period [*F*(5, 28) = 15.88, *p* < .01, η_p_^2^ = 0.33], but no significant effects for either experience level [*F*(1, 32) = 0.97, *p* = .33] or distraction [*F*(1, 32) = 0.82, *p* = .37]. Post hoc Bonferroni tests revealed a significant decline of D during both decision‐making periods of the task compared to the four watchkeeping periods [*p*<.01]. Descriptive statistics for connection density are illustrated in Figure 7. There was only one significant interaction effect in the ANOVA model, which indicated an effect between distraction and task period [*F*(5, 28) = 3.15, *p* = .03, η_p_^2^ = 0.09]. This effect is illustrated in Figure 8. Post hoc t tests revealed a significant increase in D during the fourth period of watchkeeping (watch4) for those participants in the no‐distraction group compared to the distraction group [*t*(34)=2.97, *p*<.01]. In addition, the significant trend over the six periods of the task differed for the distraction group in comparison with the main effect observed in Figure 7, that is, there was no significant difference between watch4 and either of the two decision periods (Figure 8).

**Figure 9:**
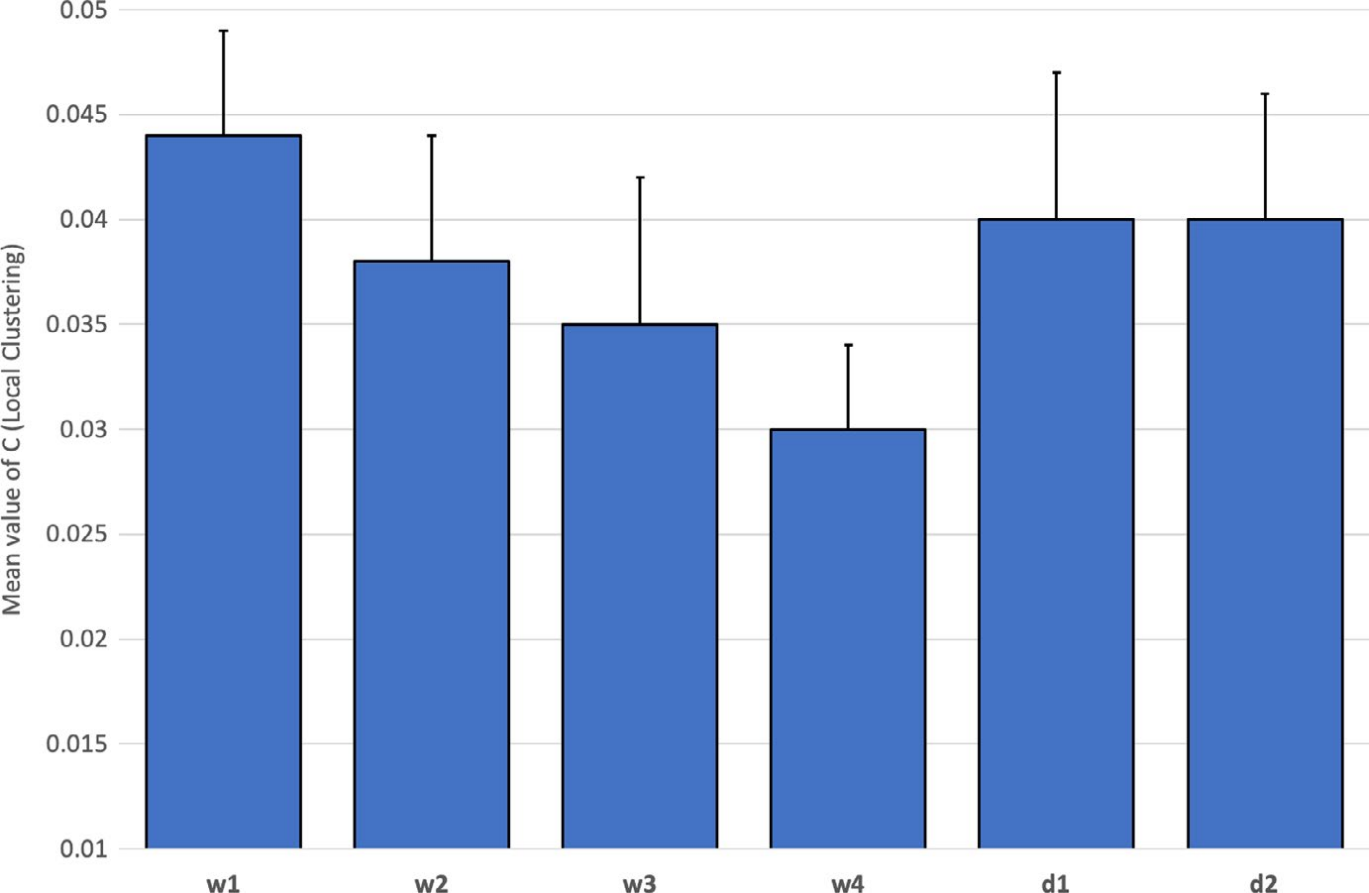
Average levels of C (clustering) with standard error across all fNIRS channels for six periods of the task (*N* = 36)

The same 2 x 2 x 6 ANOVA was conducted on the clustering coefficient (C). There were no significant main effects for either Experience or distraction, but a significant effect was found with respect to task period [*F*(5,28) = 2.60, *p* = .05, η_p_^2^ = 0.32]. Post hoc Bonferroni tests revealed that (i) C was significantly higher during decision2 compared to watch3 and watch4 (*p*<.01), (ii) C was significantly higher during decision2 compared to watch4 (*p*<.01), and (iii) C was significantly lower during watch4 compared to watch1 (*p*=.05). Descriptive statistics for C are presented in Figure 9 for the main effect of task period.

**Figure 10:**
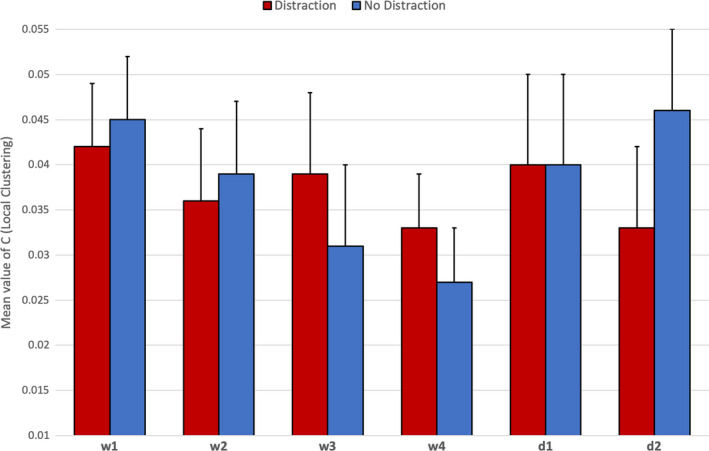
Interaction between distraction group x task periods for mean C (clustering) with standard error across all fNIRS channels for six periods of the task (*N* = 36)

This ANOVA also produced one significant interaction between distraction and task period [*F*(5, 28) = 2.79, *p* = .04, η_p_^2^ = 0.34]. Post hoc t tests revealed that the clustering coefficient was significantly lower at watch4 compared to watch1 [*t*(17)=−2.21, *p*=.04] and d2 [*t*(17)=−1.98, *p*=.05] for the no‐distraction group only. This interaction is illustrated in Figure 10.

**Figure 11:**
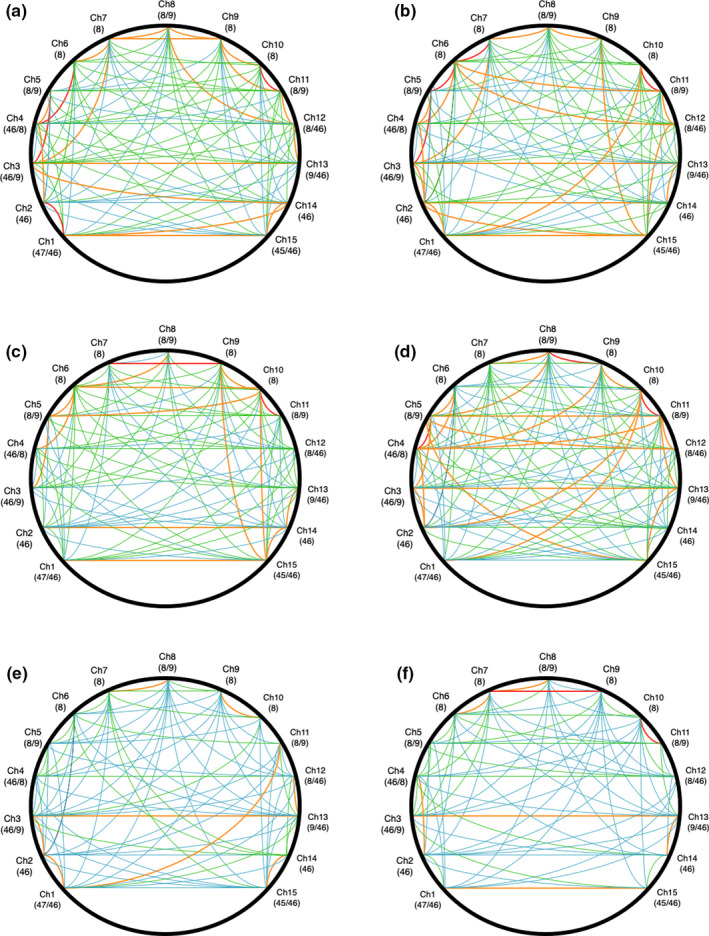
Data visualization showing relative frequency of significant connections observed in the adjacency matrices across all six task periods (N = 36): (a) watch1, (b) watch2, (c) watch3, (d) watch4, (e) decision1, and (f) decision2. Labels correspond to channels and Brodmann’s areas. Color key indicates the number of participants who exhibited a significant partial correlation coefficient for this connection, that is, red = 22 participants or more, orange = 17‐21 participants, etc

In order to understand those patterns of functional connectivity observed in the graph‐theoretic analyses, data from the binary adjacent matrices were combined into a connectome visualization based on the arc diagram (Figure 11). The purpose of this visualization was to represent the frequency of individual connections within the frontal network across the participant group as a whole; the visualization is intended to reveal which connections and patterns of connections are most prominent during all six periods of the task simulation. In Figure 11(a‐f), the color coding represents the number of participants for whom a particular connection passed the threshold, that is, partial r = 0.28 or above. A red connection denotes this connection that was observed in 22 or more of our participants, the orange lines indicate the presence of a connection for 17 ‐ 21 participants, the green for 13‐16 participants, and the blue for less than 12 participants. Hence, the color coding in Figure 11 does not correspond to the strength of each connection but rather the number of participants for whom that connection passed the threshold for a positive connection at each phase of the task simulation.

When describing the patterns of connectivity observed within each task period, we will focus on connections that were most frequently observed within our participants, that is, red and orange lines. The first period of watchkeeping (watch1) indicates a high frequency of local clustering (i.e., red/orange lines between adjacent sites) with a number of bilateral connections (Figure 11a). This pattern persists into watch2, but the frequency of bilateral connections increased (Figure 11b). During watch3 (Figure 11c), the number of adjacent and bilateral connections is observed to decline. The fourth phase of watchkeeping (Figure 11d) represented the period when participants spotted the target vessel, which was characterized by increased frequency of bilateral connections. During the first period of the decision‐making phase of the task (decision1), there is a general decrease in frequent connections (Figure 11e) with some local clustering observed at lateral areas of the montage on the left side (BA46‐BA47), the fronto‐central region (BA8/9), and a small number of bilateral connections (BA46, BA46‐BA8/9). The final part of the task (decision2) represents the period immediately prior to the participants’ performance of an evasive maneuver. During this period, the most frequent connections were clustered around the fronto‐central region (BA8) with a small number of bilateral connections at the left/right edges of the montage, for example, BA46 and BA46‐BA47 (Figure 11f).

### 3.5 Prediction of Behavior based on Functional Connectivity

A regression analysis was conducted to explore whether behavioral data could be predicted on the basis of functional connectivity metrics, for example, density and clustering. Behavioral data were obtained from two period of the task: watch4 (i.e., distance from target vessel when it was spotted) and decision2 (i.e., distance from the target vessel when course was changed). Two linear regression models were created, one for watch4 and another for decision2, each using distance as a dependent variable with density (D) and clustering (C) as independent variables.

The regression analysis conducted on data from watch4 revealed an R^2^ of 0.29 (Adj R^2^ = 0.25), which was a statistically significant model [*F*(2,34) = 6.79, *p*<.01]. Detailed inspection of the model (Table 4) revealed that increased density and clustering were both associated with the target vessel being spotted at a greater distance from the participant’s ship. From the model, density is the most reliable predictor of distance relative to clustering (Table 5). The same model was applied to comparable data from decision2; this model also reached statistical significance [*F*(2,34) = 8.07, *p*<.01] with an R^2^ value of 0.33 (Adj R^2^ = 0.29). The model revealed an inverse relationship between density and distance to the target vessel when the participant changed the course of their ship (Table 5).

**Table 5:** Results of the linear regression models with distance to Target Vessel as the dependent variable

	Watch4				Decision2			
	*t*	Std. *ß*	Partial *r*	Sig	*t*	Std. *ß*	Partial *r*	Sig
Density	3.56	0.54	0.53	<.01	‐3.47	‐0.50	‐0.52	<.01
Clustering	1.94	0.30	0.32	.06	1.37	0.20	0.23	0.18

## 4 DISCUSSION

The task simulation was divided into two major cognitive activities, watchkeeping (watch1‐4) and decision‐making/action selection (decision1‐decision2). The former represents a visual vigilance task where the participant must monitor the forward view for the appearance of other vessels. Once the target vessel was located during watch4, the participant must appraise the situation and select an appropriate course of action. During decision1, participants actively monitor the approach of the target vessel and appraise the likelihood of collision until they formulate and execute an evasive maneuver during decision2. Both categories of cognitive activity are associated with increased activation within the prefrontal cortex (PFC). The periods of watchkeeping (watch1‐watch4) require the participant to sustain attention in the absence of any overt stimuli, which is associated with activity in right lateralized regions of the dorsomedial, mid‐, and lateral prefrontal cortex (Langner & Eickhoff, 2013). With respect to the process of appraisal and action selection, the cascade model of cognitive control (Koechlin et al., 2003; Koechlin & Summerfield, 2007) argues that actions are selected on the basis of current context and past experience, and this evaluative process is localized to the caudal and lateral regions of the PFC.

Our analysis of neurovascular activation across four periods of watchkeeping (watch1‐watch4) revealed a number of statistically significant trends, specifically: (a) A decline of CBSI_HbO during watch3 and watch4 in the right lateral PFC compared to watch1 and watch2 (Figure 4), and (b) a decline of local clustering from watch1 to watch4 (Figure 9). Hence, reduced activation in the region of the right PFC was accompanied by a general reduction of local clustering across the whole montage. With respect to the visualization of connectivity (Figure 11), the first and second periods of watchkeeping (Figure 11a,b) are characterized by high frequency of connections across the montage, particularly on the left lateral channels 1‐5 and significant levels of bilateral connectivity. During watch3 (Figure 11c), participants have been monitoring an empty ocean for approx. 14.5 minutes and we observed bilateral activation in the central (BA8) and lateral (BA46) regions with localized connections on the right hemisphere, for example, BA46 and BA8‐BA46. Activation of right PFC during vigilant attention has been reported in a number of neuroimaging studies (Langner & Eickhoff, 2013; Parasuraman et al., 1998) and damage to this area of the brain associated with a diminished capacity for sustained attention (Swick & Knight, 1998). The reduction of local clustering observed during watch3 receives circumstantial support from an existing fNIRS study where reduced functional connectivity was associated with the performance of a sustained attention task (Wang et al., 2016).

The final period of watchkeeping (watch4) marked a transition from vigilance to action selection when participants had located the target vessel. While neurovascular activation during watch4 was characterized by reduced activation in the right lateral PFC (Figure 4) and a decline of local clustering (Figure 9), it was also marked by a noticeable increase in bilateral connections, that is, relative to the previous period watch3 (Figure 11c). In comparing the period of action selection with the vigilance phase, we noted a significant decline of connection density (Figure 7) and increased local clustering (Figure 9). It has been argued that the rostral area of the lateral PFC (BA 46) is crucial for the integration of previous experience with the current context during action preparation (Domenech & Koechlin, 2015; Koechlin & Summerfield, 2007); therefore, increased activation in the right lateral PFC (Figure 4) is broadly consistent with this explanation.

Concerning the pattern of functional connectivity observed in Figure 7, the primary distinction between watch4 and decision1 was a significant decrease in the overall number of connections. This pattern is supported by the visualization presented in Figure 11, that is, fewer orange and red connections appear in decision1 and decision2 compared to watch1‐4 (Figure 11). This decline was particularly noticeable for bilateral connectivity as participants transitioned from watch4 to decision1 (Figure 11). Closer inspection of Figure 11e indicated that the process of action selection that was initiated during decision1 was associated with a small number of localized connections in the central area (BA8) and left/right lateral channels (BA46, BA47); we also observed a limited number of bilateral connections, for example, Ch3‐Ch13 and Ch1‐Ch11. During decision2 when participants actually executed an evasive control maneuver via the helm, we see a high number of bilateral activations at BA8 (Figure 11f). It should also be noted that BA8 lies very close to the premotor cortex (BA6) and preparation of the motor response may explain the high levels of connectivity in the fronto‐central region that were observed during this period. This period was also associated with highly localized bilateral connectivity in the regions of BA46, BA47, and BA45 (Figure 11f). This observed pattern of bilateral activation at BA46 is associated with Episodic control over action selection in previous experimental work, that is, guidance of action selection that can be attributed to past experience (Koechlin et al., 2003). The same model predicts that the caudal region of the lateral PFC (BA9, BA45) is associated with Contextual control where stimulus–response associations are selected on the immediate context in which the stimulus (e.g., target vessel) occurs (Koechlin & Summerfield, 2007), that is, Figure 11e. The pattern of persistent bilateral connectivity and increased activity in the lateral PFC may represent a trade‐off between the exploitation of previous experience and exploration of the immediate context, as participants assessed the approach of a target vessel and formulated an evasive maneuver (Koechlin, 2016).

The purpose of the regression analyses was to explore the relationship between functional connectivity in the PFC and behavioral outcomes during the task simulation. If the PFC exerts a top‐down influence on fundamental psychological processes, such as sustained visual attention and action selection, it is reasonable to expect a degree of correlation between neurophysiological activation in the PFC and performance outcomes. Two linear regression models were constructed to predict distance from the target vessel when it was (a) spotted and (b) when participants performed a course change to avoid collision (Table 5). Both models were statistically significant, functional connectivity metrics accounted for approximately a third of variance observed in the performance data, a figure that was substantially higher than we anticipated. The watch4 model revealed that density and local clustering were both positively associated with distance to target vessel when it was spotted by participants; however, this relationship was strongest for connection density (Table 5). By contrast, we found an inverse relationship between connection density and the safety margin in the decision2 model (Table 5), that is, reduced density was associated with greater distance to target vessel when an evasive maneuver was performed. Both models reinforce trends observed in Figure 7, that is, increased connection density during vigilance and a significant decline of density during action selection. The regression models also confirm an association between measures of functional connectivity in the PFC and performance outcomes in an applied, safety‐critical scenario.

Participants were divided into two groups based on previous seafaring experience. Our analysis of behavioral data revealed that experienced participants made an evasive maneuver at a greater distance from the target vessel compared to those participants who were less experienced (Table 3). Therefore, as one might expect, experienced seafarers respond with greater efficiency to a collision avoidance scenario, presumably due to a superior ability to assess and respond to a safety‐critical situation that was informed by previous experience. Also, analyses of the subjective workload data revealed that experienced participants perceived the task simulation to be less effortful and appraised their own performance positively in comparison with inexperienced participants (Table 4). It was hypothesized that neurovascular activation would be lower for experienced participants due to greater neural efficiency (Causse et al., 2017; Di Domenico et al., 2015; McKendrick et al., 2014), but there was no evidence in the current study to support this position. The analysis of fNIRS data indicated that experienced participant exhibited higher levels of CBSI_HbO during watch4 at the right lateral PFC (Figure 6). This finding was notable as higher activation of the PFC during watch4 may represent the influence of top‐down control over visual attention (Paneri & Gregoriou, 2017) as experienced participants sought to resolve uncertainty over the presence and location of the target vessel. However, it should be noted that this pattern of neurovascular activation did not result in earlier detection of the target vessel by experienced participants (Table 2).

Half of the participants were required to perform a distraction task of reporting ship position, which was repeated approximately every three minutes. This additional task increased activation at the right lateral PFC during the two decision‐making periods of the task (Figure 5), presumably due to the multiple demands of simultaneously monitoring the approach of the target vessel, formulating change of course, and monitoring the current position of their own ship in order to cue the next verbal report. It should be noted that the average duration of each decision‐making phase was significantly shorter than the watchkeeping phases, for example, 142s compared to 292.5s. Participants received 1.65 distractions per watchkeeping phase compared to 0.78 distractions in each phase of the decision‐making part of the task; hence, the influence of distraction was not equivalent across both phases of the simulated task. The effects of the distraction task on functional connectivity indicated: (a) a reduction of connection density during watchkeeping, which reached statistical significance during watch4 (Figure 8), and (b) the absence of the significant decline in local clustering over the watchkeeping phase (watch1‐4) that characterized participants in the nondistraction condition (Figure 10). Given that decreased connection density was associated with higher cognitive load during the decision‐making phase (Figure 7), it could be argued that the distraction inflated cognitive demand for those participants during a monotonous vigilance task, which subsequently reduced connection density. Similarly, the decline of local clustering during watchkeeping did not occur for the distraction group because these individuals received an additional level of cognitive demand, which may have helped to sustain attention. It was hypothesized that the introduction of a distraction task would degrade performance concerning increasing response latencies, with respect to spotting the target vessel and making an evasive maneuver, but no evidence was found to support this prediction.

The current study was not without limitations and potential confounds. The task simulation was highly simplistic and designed to facilitate collection of neurophysiological data. It could be argued that the ecological validity of the simulation was compromised by our desire to reduce artifacts in the fNIRS data. For example, the task simulation failed to accommodate any aspect of teamwork, which is the more common operational situation on the bridge of a large ship; besides, watchkeeping duty is often part of a multitasking activity that includes monitoring weather conditions and running communications tasks. The duration of the vigil in the first part of the simulation was less than twenty minutes, and it is acknowledged that extending the period of watchkeeping would have improved the ecological validity of the study. Also, the decision not to utilize the 360° field of view capability in the bridge simulator (in order to minimize the influence of physical artifacts in the fNIRS signal) was problematic, as it enormously simplified and artificially constrained the challenge of the vigilance task in a maritime environment. Our decision to seat participants at the helm of the vessel was also uncharacteristic of the bridge environment; participants were seated to minimize those systemic influences on the fNIRS signal that were likely to occur if they were standing and ambulatory. The fNIRS apparatus utilized in the current study was problematic because we were unable to fully account for systemic influences on the fNIRS signal. The montage used in the study did not include “short‐leads” that can be utilized to quantify and correct the systemic contribution of blood flow in the superficial tissues to the neurovascular response; see Pfeifer et al. (2018) for a recent discussion of signal processing protocols using fNIRS. The graph‐theoretic measures used in the study were based upon a matrix of partial correlations, which has limitations as an index of connectivity; specifically, they only incorporate bivariate association and cannot account for relationships between cortical sites that occur with time lags (Baccalá & Sameshima, 2001). Alternative approaches, such as dynamic causal modeling (Stephan & Friston, 2010) and Granger causality modeling (Seth et al., 2015), do not suffer from these shortcomings and may represent superior measures of fNIRS connectivity, for example, Sun et al. (2019)

Head movement represents a significant source of potential confounding for the fNIRS signal, particularly in combination with a frontal montage and participants being able to tilt the head longitudinally, which could potentially elevating blood flow and CBSI_HbO when the head is tilted downwards (Cui et al., 2015). This confound is particularly of concern when participants performed the distraction task and were forced to switch their gaze from the ECDIS display and the forward view. It could be argued that increased CBSI_HbO observed during decision1 and decision2 for the group performing the distraction task (Figure 5) may have been strongly influenced by repeated up/down head movements. In hindsight, the placement of an accelerometer on the head of the participant would permit these episodes to be identified, corrected, or removed during the data analyses. The fNIRS montage was also limited in at least two aspects: (1) The montage sacrificed coverage of the cortex for resolution of the PFC, and an increased number of channels in other locations (e.g., central, parietal, and occipital) would have provided greater context, particularly for identifying unique patterns of frontal connectivity; and (2) the spatial placement of the optodes was based upon the 10/20 system and should be regarded as highly approximate given differences in skull size, shape, etc. In order to improve the fidelity of the study while enhancing ecological validity, it is essential to compensate for the influence of real‐world factors on the fNIRS signal (Ayaz et al., 2013) and derive signal processing protocols (Kamran et al., 2016) that permit this method to be utilized with confidence in naturalistic settings (Pinti et al., 2019).

The current study introduced several issues that would benefit from further study and investigation. Bridge simulators are generally used in the maritime industry for purposes of training, and there is little standardization of scenarios. If we wish to use these facilities to study maritime operations systematically, it is crucial to develop simulator scenarios that (a) incorporate real‐world complexity, (b) mimic operations and procedures that are associated with errors and accidents in the real world, and (c) capture demands concerning multiple individuals working within teams, including the inherent hierarchy within those teams. It is also crucial to design scenarios within the simulation that are fully representative with respect to capturing the duration and variety of demands that are encountered during actual maritime operations. The incorporation of techniques like fNIRS into this type of task simulation represents the first step in a trajectory of research within the maritime domain that encompasses: (a) hyperscanning research to assess teamwork, that is, collecting neurophysiological signals simultaneously from two individuals working collaboratively in a safety‐critical environment (Toppi et al., 2016), and (b) the utilization of fNIRS as an implicit measure of operator attention during system automation, that is, when no behavioral response is required from the operator (Verdière et al., 2018).

Concerning future work and developments in this area of research, some aspects of the current study deserve further comment. The field of neuroergonomics (Ayaz & Dehais, 2019; Parasuraman & Rizzo, 2008) is based upon an implicit assumption that the integration of neuroscientific methods into human factors research will enhance our understanding of safety‐critical performance (Hancock, 2019; Parasuraman, 2003). In order for this initiative to deliver on this potential, neuroscientific models must be explored within the context of real‐world behavior, for example, such as Koechlin’s model of cognitive control in the current work (Koechlin & Summerfield, 2007). This process of neuroscientific inference can be challenging, and application of these methods into applied, operational environments presents an additional level of complexity concerning data interpretation. Secondly, for neuroscientific methods to have sufficient relevance for human factors research, neurophysiology must make a contribution to our understanding of safety and human–machine interaction that is both significant and unique. Measuring the brain at work is relatively straightforward with modern sensor technology, but imbuing these methods with explanatory power and predictive capability remains a challenge for this emerging field. The current study demonstrated a significant association between functional connectivity and behavioral outcomes in a safety‐critical task, which provides an empirical demonstration of the potential utility of neurophysiological assessment in operational settings. If this finding can be replicated and expanded, that is, see Ayaz et al. (2019) for a similar initiative, neuroscientific methods will enhance our understanding of safety‐critical behaviors and deliver practical benefits for operator training, design of technology, and operational protocols.

To conclude, the current study measured neurovascular activation and functional connectivity in the context of ship bridge operations. Increased activation of the right lateral PFC, reduced connection density, and a higher level of local clustering across a frontal montage of fifteen channels were associated with action selection in comparison with the earlier watchkeeping period of vigilant attention. Activity in the right lateral PFC and the level of local clustering declined across the watchkeeping period for participants. The study also demonstrated a significant association between metrics of frontal connectivity (i.e., connection density) and behavioral responses to a safety‐critical scenario.

## Conflict of Interest

The authors have no conflicts of interest to declare.

## Author Contribution

ShF contributed to study planning, study design, preparation of materials, design of simulation, pilot trials, running the data collection, data analyses, writing the manuscript, and proofing the manuscript. EBD contributed to study planning, study design, design of simulation, and proofing the manuscript. JZ contributed to proofing the manuscript. AB contributed to study planning, study design, preparation of materials, design of simulation, and pilot trials. JW contributed to study planning, study design, preparation of materials, design of simulation, and pilot trials. ZY contributed to study planning, study design, and proofing the manuscript. XY contributed to proofing the manuscript. JW contributed to proofing the manuscript. SF contributed to study planning, study design, preparation of materials, design of simulation, pilot trials, data analyses, writing the manuscript, and proofing the manuscript.

### Peer Review

The peer review history for this article is available at https://publons.com/publon/10.1002/brb3.1910.

## Data Availability

The data that support the findings of this study are available on request from the corresponding author. The data are not publicly available due to privacy or ethical restrictions.
